# Integrated Stress Response as a Therapeutic Target for CNS Injuries

**DOI:** 10.1155/2017/6953156

**Published:** 2017-04-27

**Authors:** Lorenzo Romero-Ramírez, Manuel Nieto-Sampedro, M. Asunción Barreda-Manso

**Affiliations:** ^1^Unidad de Neurología Experimental, Hospital Nacional de Parapléjicos (SESCAM), Finca la Peraleda s/n, 45071 Toledo, Spain; ^2^Departamento de Neurobiología Funcional y de Sistemas, Laboratorio de Plasticidad Neural, Instituto Cajal (CSIC), Avenida Doctor Arce 37, 28002 Madrid, Spain; ^3^Laboratorio de Endocannabinoides y Neuroinflamación, School of Biosciences, Universidad Francisco de Vitoria, 28223 Pozuelo de Alarcón, Madrid, Spain

## Abstract

Central nervous system (CNS) injuries, caused by cerebrovascular pathologies or mechanical contusions (e.g., traumatic brain injury, TBI) comprise a diverse group of disorders that share the activation of the integrated stress response (ISR). This pathway is an innate protective mechanism, with encouraging potential as therapeutic target for CNS injury repair. In this review, we will focus on the progress in understanding the role of the ISR and we will discuss the effects of various small molecules that target the ISR on different animal models of CNS injury.

## 1. Introduction

CNS injuries are a diverse group of disorders that include spinal cord injury (SCI), traumatic brain injury (TBI), and stroke. Although different in etiology, CNS injuries trigger shared processes such as disruption of the blood-brain barrier (BBB) [1, 2] or the blood-spinal cord barrier (BSCB) [3] that facilitate the extravasation of blood substances and cells into the CNS parenchyma and vice versa, excitotoxicity [4–6], and hypoxia/ischemia [5, 7], increase the inflammatory response activated after injury [5, 7, 8], and spread the initial cell death due to the injury to other CNS areas, with added detrimental effects.

This acute neuroinflammatory response causes the activation of glial cells (mainly astrocytes and microglia) that counterbalance the changes in tissue homeostasis [9, 10]. Glial cells show enhanced migration into the injured site [11, 12] and release inflammatory mediators including pro- and anti-inflammatory cytokines and chemokines [9, 10]. The proinflammatory mediators induce the activation and recruitment of leukocytes to the inflammation site in the CNS parenchyma [10, 13]. Activated microglia (CNS resident macrophages [14]), as well as infiltrating neutrophils [15] and monocytes [16], phagocyte debris from damaged tissue and dead cells. Activated astrocytes and mesenchymal cells (meningeal fibroblasts [17], perivascular fibroblasts [18], and pericytes [19]) migrate to the injury site and attempt to restore the disrupted BBB or BSCB, secreting extracellular matrix proteins that induce a new* glia limitans*, called glial scar [17, 20]. Both phagocytosis of dead cells and glial scar formation attempt to reduce inflammation and restore tissue homeostasis [21, 22]. However, both processes might have detrimental effects. Activated microglia/macrophages may phagocyte living cells increasing tissue loss [23] and the glial scar is one of the main obstacles to axonal regeneration after injury [24]. If these processes cannot restore homeostasis, the inflammatory response is maintained long after injury. This state of chronic neuroinflammation increases further the loss of white and grey matter that characterizes many CNS pathologies [25, 26].

## 2. Integrated Stress Response in CNS Injury

**Figure 1:**
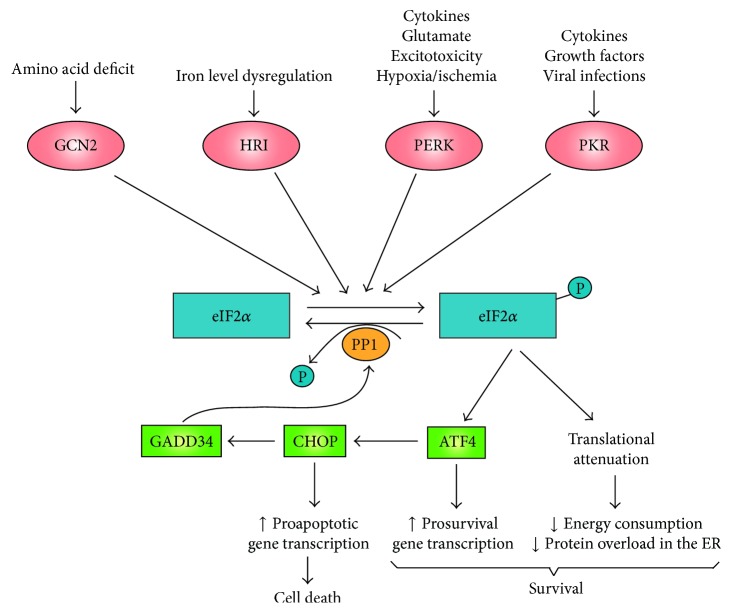
*The integrated stress response is sensitive to numerous stimuli activated after CNS injury*. A diverse group of kinases such as GCN2, HRI, PERK, and PKR induce the phosphorylation of eIF2*α* in response to various stimuli resulting in translational attenuation to overcome the accumulation of misfolded or unfolded proteins in the ER and retrieve proteostasis. Phosphorylated eIF2*α* (eIF2*α*-P) enhances the translation of the transcription factor ATF4 that induces the transcription of prosurvival genes as well as the transcription factor CHOP. Both ATF4 and CHOP cause upregulation of GADD34 expression, which forms a complex with PP1, inducing the dephosphorylation of eIF2*α*-P and hence retrieving translation. When protein homeostasis is not restored (indicating cellular damage), CHOP increases the transcription of proapoptotic genes, inducing cell death. ATF4, activating transcription factor 4; CHOP, C/EBP homolog protein; eIF2*α*, eukaryotic translation initiation factor 2*α*; GADD34, growth arrest and DNA-damage inducible 34; GCN2, general control nonderepressible 2; HRI, haem-regulated inhibitor kinase; P, inorganic phosphate; PERK, protein kinase RNA-like endoplasmic reticulum kinase; PKR, double-stranded RNA-activated protein kinase; PP1, protein phosphatase 1.

CNS injury can cause oxygen-glucose deprivation, amino acid deprivation, glutamate excitotoxicity, oxidative stress, and the release of cytokines. These may affect protein synthesis at the endoplasmic reticulum (ER), inducing the accumulation of misfolded or unfolded proteins therein [27–30] (Figure 1). The integrated stress response (ISR) is activated as a cytoprotective mechanism [31], to maintain cellular protein-folding homeostasis (also known as proteostasis) [32]. A diverse group of kinases (Figure 1) act as sensors for different stressors activating the ISR, through the phosphorylation of the alpha subunit of the eukaryotic translation initiation factor 2 (eIF2*α* to eIF2 *α*-P) on Serine 51 [32]. Phosphorylated eIF2*α* binds to eukaryotic translation initiation factor 2B (eIF2B). This inhibits its activity and attenuates protein translation while inducing the translation of activating transcription factor 4 (ATF4, also known as CREB2) and other selected genes [30]. ATF4 upregulates the expression of chaperones (e.g., Bip, also known as GRP78 [33, 34]) that help to reduce protein accumulation in the ER. It also promotes the expression of prosurvival factors involved in amino acid metabolism and oxidative stress resistance [30, 35]. Moreover, ATF4 increases the expression of transcription factor CHOP (C/EBP homologous protein, also known as DDIT3, DNA-damage-inducible transcript 3). Both ATF4 and CHOP upregulate the expression of GADD34 (growth arrest and DNA-damage inducible 34; also known as protein phosphatase 1 regulatory subunit 15A or PPP1R15A). GADD34 is a regulatory cofactor of the catalytic subunit of protein phosphatase 1 (PP1) that directs the dephosphorylation of eIF2*α* restoring protein synthesis [36]. Should the restoration of proteostasis fail, the transcription factor CHOP would cause an increase in the expression of genes involved in apoptosis, executing the damaged cell [37].

In addition to the stress-inducible GADD34-PP1 complex, CReP (constitutive repressor of eIF2*α* phosphorylation, also known as PPP1R15B) forms a complex with PP1 that constitutively dephosphorylates eIF2*α* [38].

## 3. CNS Injury Therapeutics Based on the Integrated Stress Response 

CNS injury causes the loss of white and grey matter with detrimental effects [25, 26]. After the initial cell death and tissue destruction caused by the injury, numerous mechanisms are activated spreading both tissue damage and cell death (like excitotoxicity [4–6], hypoxia/ischemia [5, 7, 8], and so forth). CNS injuries enhance the ISR pathway as a cytoprotective mechanism [27, 39], suggesting that the ISR might be a therapeutic target for CNS injuries. Several small molecules, such as salubrinal [40], guanabenz [41], and Sephin1 [42], that enhance ISR have shown cytoprotective effects.

### 3.1. Salubrinal

**Figure 2:**
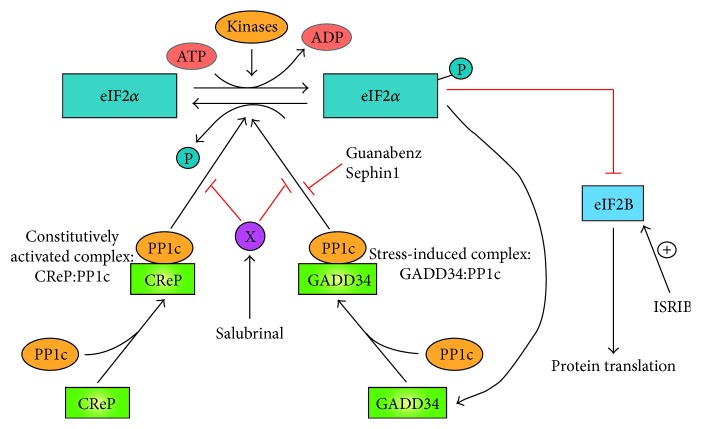
*Pharmacological regulation of the integrated stress response (ISR).* The translation initiation factor eIF2*α* is phosphorylated (eIF2*α*-P) by a diverse group of kinases. Phosphorylated eIF2*α* represses eIF2B inducing translation attenuation as well as activation of a pathway that induces GADD34 forming a complex with PP1c (GADD34:PP1c). This stress-activated complex induces the desphosphorylation of eIF2*α*-P to restore proteostasis. A constitutively activated complex (CReP:PP1c) dephosphorylates eIF2*α*-P under basal conditions. Salubrinal inhibits indirectly the activity of both GADD34:PP1c and CReP:PP1c complexes through an unknown target (X). However, Guanabenz and Sephin1 only inhibit the activity of GADD34:PP1c complex. ISRIB overcomes the attenuation of translation induced by eIF2*α*-P, activating eIF2B downstream of eIF2*α*. ADP, adenosine diphosphate; ATP, adenosine triphosphate; CReP, constitutive repressor of eIF2*α* phosphorylation; eIF2*α*, eukaryotic translation initiation factor 2*α*; eIF2*α*B, eukaryotic translation initiation factor 2B; GADD34, growth arrest and DNA-damage inducible 34; ISRIB, integrated stress response inhibitor; P, inorganic phosphate; PP1c, protein phosphatase 1 catalytic subunit.

Salubrinal is a small molecule discovered in a high-throughput screening of compounds with cytoprotective effect on ER stress-induced cell death [40]. Although the target for salubrinal is unknown, it maintains eIF2*α* highly phosphorylated, reducing protein synthesis at the ER [40]. Salubrinal inhibits the activity of both CReP-PP1 constitutive complex and GADD34-PP1 stress-inducible complex (Figure 2).

Salubrinal has neuroprotective effects in animal models of CNS injury, such as a rat brain excitotoxicity [43], cerebral ischemia/reperfusion [44, 45], and chronic intermittent hypoxia [46]. It is neuroprotective also in mouse models of sleep apnea [47], traumatic brain injury [48, 49], and cortical stab injury [50]. Moreover, salubrinal protects oligodendrocytes, reducing demyelination and improving functional recovery after spinal cord injury [51]. The prosurvival effects of salubrinal after CNS injury are mediated by decreasing the ER stress response [43, 45, 48–50]. Limiting energy consumption is achieved under pathological conditions by diminishing ER protein overload [52].

Recent evidence suggests that salubrinal treatment may trigger additional cytoprotective pathways by upregulating the expression of platelet-derived growth factor subunit B (PDGF-B) [53–56] in neurons close to the injury [50]. After a cortical stab injury, salubrinal also helps in restoring the integrity of the blood-brain barrier (BBB) by inducing an increase of fibronectin expression and a reduction of the activation of microglia/macrophages [50]. A rapid restoration of the BBB integrity following injury is essential to restore homeostasis [57]. Should this process be delayed or impeded, blood substances and leukocytes would continue diffusing into the CNS, triggering an additional inflammatory process. This would extend the initial injury, strengthening the so-called secondary neuronal loss, with increased detrimental effects [58]. PDGF-B is a mitogen for pericytes and mesenchymal cells [59], inducing both proliferation and production of fibronectin [60]. The release of PDGF-B in the proximity of the lesion site might help to close the injury, accelerating BBB integrity restoration [50]. Moreover, in a rat model of global cerebral ischemia, salubrinal reduced the levels of matrix metalloprotease 9 (MMP-9), as well as of the injury-induced cell adhesion molecules ICAM-1 and VCAM-1 [46]. MMP-9 is a typical marker of BBB impairment [61, 62] and both ICAM-1 and VCAM-1 are involved in leukocyte migration into the injured CNS [63]. In conclusion, these results support the restorative effect of salubrinal treatment on BBB integrity after injury [50, 64]. The reduction of both microglia activation and blood monocyte infiltration into the injury site may diminish inflammation, reducing neuronal death and tissue loss after injury [50, 64].

Salubrinal may show additional neuroprotective and anti-inflammatory effects by inhibiting the transcription factor NF*κ*B pathway [65, 66]. Indeed, salubrinal treatment reduced both neuronal cell death and microglia activation by *β*-amyloid (A*β*) [65] inhibiting the NF*κ*B pathway. The authors propose that these effects are mediated by the inhibition of IkB kinase (IKK) activation, consequently reducing the degradation of the NF*κ*B repressor IkB [65]. Another article reported that both salubrinal and guanabenz selectively reduce TNF*α* but not IL-1*β*-induced activation of NF*κ*B [66]. This work concluded that the effect occurred upstream of transforming growth factor/beta-activated kinase 1 (TAK1) and was independent of eIF2*α* [66]. These results suggest that both salubrinal and guanabenz may regulate also the phosphorylation of other targets [67].

Following severe CNS injuries, glial cells (mainly astrocytes) and mesenchymal cells (fibroblasts and pericytes) attempt to restore the disrupted BBB, by secreting extracellular matrix proteins and forming a new* glia limitans,* called the glial scar [17]. Although glial scar formation has some beneficial effects [68], it is also one of the main obstacles to axonal regeneration after injury [24]. The major axon regenerative growth inhibitors in the glial scar are the chondroitin sulfate proteoglycans (CSPGs) [69]. The neuritogenic effects shown by salubrinal* in vitro* are due to the inhibition of the expression in glial cells of CSPGs and other profibrotic proteins, such as CTGF [70]. In addition to the effect on protein translation, salubrinal reduced the mRNAs for CSPGs and CTGF [52]. These processes may work together to reduce protein overload in the ER, speed up proteostasis, and increase cell survival after CNS injury.

These results suggest that salubrinal could be a good candidate for pharmacological therapy of CNS injuries. Detrimental effects of salubrinal treatment have been reported in pancreatic beta cells exposed to fatty acids [71] and in the neuroprotection induced by preconditioning, in a rodent model of permanent ischemia [72]. Since the mechanism of action of salubrinal is unknown, these adverse effects might be due to inhibition by salubrinal treatment of both constitutive and stress-induced phosphorylation of eIF2*α* [73], or an off-target effect.

### 3.2. Guanabenz and Sephin1

Guanabenz is an agonist of the *α*2-adrenergic receptor and it is a prescription drug to treat hypertension [74]. In addition, guanabenz selectively inhibits stress-induced dephosphorylation of eIF2*α* by the GADD34:PP1 complex, without affecting the activity of the constitutively activated CReP:PP1 complex (Figure 2) [41]. Guanabenz showed beneficial effects in a rodent model of multiple sclerosis, suggesting that the inhibition of GADD34:PP1 complex alone was enough to improve mouse recovery [75]. In particular, guanabenz was cytoprotective for oligodendrocytes and reduced demyelination [75]. These positive results have led to a phase I clinical trial to determine the safe dose of guanabenz for multiple sclerosis patients [73].

In contrast to multiple sclerosis, guanabenz treatment in rodent models of CNS injuries showed contradictory effects. Thus, it was cytoprotective for oligodendrocyte precursor cells (OPC)* in vitro*, but it did not enhance functional recovery in a mouse model of SCI [76]. In a rat model of TBI, guanabenz was neuroprotective, reduced cortical contusion, and decreased hipocampal cell damage, attenuating motor, learning, and memory deficits after TBI [77]. However, the dose of guanabenz in TBI experiments was five times higher (5 mg/Kg) than in SCI experiments (1 mg/Kg). In mice, a dose of guanabenz in that range (1–5 mg/Kg) has side effects manifested by a dose-dependent decrease in rotarod performances [42]. These side effects may be due to the effect of the drug on adrenergic receptors, lowering mice blood pressure. TBI mice treated with the FDA-approved dose range (0.5 mg/Kg) had beneficial effects, but not all the benefits observed with the higher dose [77]. Guanabenz is used to lower blood pressure in patients with hypertension. A high dose of guanabenz has side effects, including drowsiness and coma [78]. These results hamper the possible use of guanabenz for treatment of CNS injuries.

Sephin1 is a guanabenz derivative with selective inhibitory effect on stress-induced dephosphorylation of eIF2*α* by the GADD34:PP1 complex (Figure 2), without *α*2-adrenergic activity [42]. Sephin1 showed beneficial effects on animal models of two protein-misfolding diseases, Charcot-Marie-tooth 1B (CMT1B) and Amyotrophic lateral sclerosis (ALS), without any side effect in chronic treatments [42]. In particular, Sephin1 treatment (1–5 mg/Kg) did not decrease mice performance in rotarod, or in the Morris water maze, that tests spatial learning [42]. In fact, mice treated with Sephin1 prevented motor deficits in untreated mice of MPZ^mutant^ (a mouse model of CMT1B) and SOD1^mutant^ (a mouse model of ALS).

It is unknown whether Sephin1 treatment has beneficial effects on CNS injuries. The desirable properties of Sephin1, such as specificity for stress-induced complex and the reduced *α*2-adrenergic activity, deserve a study to determine its therapeutic potential for CNS injuries.

## 4. Conclusions 

The integrated stress response (ISR) is a cytoprotective mechanism induced in CNS injuries. The enhancement of ISR pathway by the small molecule salubrinal is neuroprotective and neuritogenic and helps to restore BBB integrity after injury. A new generation of small molecules that enhances the ISR pathway, such as Sephin1, is currently under investigation to determine their potential for the treatment of CNS injuries. Preliminary results indicate that the enhancement of the ISR pathway is a promising therapeutic target for CNS injuries.
